# Modeling the Effects of Cu Content and Deformation Variables on the High-Temperature Flow Behavior of Dilute Al-Fe-Si Alloys Using an Artificial Neural Network

**DOI:** 10.3390/ma9070536

**Published:** 2016-06-30

**Authors:** Mohammad Shakiba, Nick Parson, X.-Grant Chen

**Affiliations:** 1 Department of Applied Science, University of Québec at Chicoutimi, Saguenay, QC G7H 2B1, Canada; xgrant_chen@uqac.ca; 2 Arvida Research and Development Centre, Rio Tinto Alcan, Saguenay, QC G7S 4K8, Canada; nick.parson@riotinto.com

**Keywords:** 1xxx aluminum alloys, hot deformation, flow stress prediction, artificial neural network modeling, sensitivity analysis

## Abstract

The hot deformation behavior of Al-0.12Fe-0.1Si alloys with varied amounts of Cu (0.002–0.31 wt %) was investigated by uniaxial compression tests conducted at different temperatures (400 °C–550 °C) and strain rates (0.01–10 s^−1^). The results demonstrated that flow stress decreased with increasing deformation temperature and decreasing strain rate, while flow stress increased with increasing Cu content for all deformation conditions studied due to the solute drag effect. Based on the experimental data, an artificial neural network (ANN) model was developed to study the relationship between chemical composition, deformation variables and high-temperature flow behavior. A three-layer feed-forward back-propagation artificial neural network with 20 neurons in a hidden layer was established in this study. The input parameters were Cu content, temperature, strain rate and strain, while the flow stress was the output. The performance of the proposed model was evaluated using the K-fold cross-validation method. The results showed excellent generalization capability of the developed model. Sensitivity analysis indicated that the strain rate is the most important parameter, while the Cu content exhibited a modest but significant influence on the flow stress.

## 1. Introduction

1xxx series wrought aluminum alloys are used in a wide range of applications and product forms, such as foil and strips for packaging and heat-exchanger tubing, cable sheathing and fin stock, where excellent formability, corrosion resistance and electrical and thermal conductivity are required [1,2,3]. Typically, the levels of iron and silicon in a specific alloy are controlled to provide the required performance characteristics, such as strength, formability or corrosion resistance, which resulting in many commercial variants within a given AA specification. The effects of iron and silicon levels on hot workability in 1xxx alloys were reported in our previous work [4]. Increasing both iron and silicon contents generally increases the high-temperature flow stress, which can negatively impact the hot workability [4,5,6,7]. For example, the extrusion speed and corresponding productivity of thin-wall tubing can be reduced. Thus, there is a trade-off between room temperature mechanical properties and hot workability. An alternate approach to increase room temperature strength is to add copper for solution strengthening, as is the case in the widely utilized AA1100 alloys. In the current work, the impact of copper content on hot workability is assessed with the long-term view of optimizing 1xxx alloy design for both strength and hot processability.

The flow behavior of aluminum alloys during hot deformation is complex. The work hardening and dynamic softening mechanisms are both significantly affected by a number of factors, such as chemical composition, forming temperature, strain rate and strain [8,9]. Constitutive models are extensively employed to describe the correlation between flow stress, strain, strain rate and temperature during hot deformation. In addition to the deformation parameters, the alloy chemical composition also has a significant impact on the high temperature flow behavior of aluminum alloys. Therefore, it is of great interest to develop a model that represents the relationship between chemical compositions, deformation variables and flow stress. This model can be employed to optimize the chemical composition of dilute Al-Fe-Si-Cu alloy as well as process parameters to obtain deserved strength and processability. Constitutive models are either analytical [10,11,12] or phenomenological [13,14,15]. Analytical constitutive models are based on the physical aspects of a material’s behavior and require comprehensive understanding of the underlying mechanisms that control the materials’ deformation. In addition, there are many independent parameters in analytical constitutive equations that require experimental determination. These features make this type of model difficult to apply. An alternative approach would be adapting a phenomenological model based upon empirical observations. However, these models are typically restricted to certain processing domains where a specific deformation mechanism operates and the accuracy of the flow stress predicted by these regression methods is low.

In recent years, artificial neural networks (ANNs) have provided a fundamentally different approach for material modeling and material processing control techniques [16]. The most important advantage of ANNs is that they do not require postulation of a mathematical model at the outset or the identification of its parameters. ANNs learn from examples and recognize patterns in a series of input and output data without the need for any prior assumptions about their nature and interrelations [17,18]. Recently ANNs have been successfully applied to model the high-temperature flow behavior of stainless steels [18], aluminum alloys [19,20], magnesium alloys [21], titanium alloys [22,23] and Al-base metal matrix composites [24]. However, to date, no ANN model has been developed to simultaneously include the effect of both chemical composition and deformation variables in aluminum alloys.

In the present study, the capability of the ANN approach to predict the high-temperature flow behavior of Al-0.12Fe-0.1Si-Cu alloys was examined as a function of chemical composition and process parameters. An ANN model has been proposed to predict the flow behavior of Al-0.12Fe-0.1Si alloys with various levels of Cu addition (0.002–0.31 wt %) under different deformation conditions. Sensitivity analysis was carried out to quantify the relative importance of Cu addition and individual deformation variables on the flow stress.

## 2. Experimental Procedures

Al-0.12Fe-0.1Si alloys with different Cu contents ranging from 0.002% to 0.31% were investigated (all alloy compositions in this study are given in wt % unless otherwise indicated). Materials were prepared from commercially pure aluminum (99.7%), Al-50%Si and Al-50%Cu master alloys. Table 1 provides the chemical compositions of the experimental alloys used. For each composition, approximately 3 kg of material was melted in an electrical resistance furnace and then cast into a rectangular permanent steel mold. Prior to casting, the melts were grain-refined by the addition of 0.015% Ti in the form of an Al-5Ti-1B master alloy. The cast ingots of these alloys were homogenized at 550 °C for 6 h, and then water quenched to room temperature.

Cylindrical samples (10 mm diameter and 15 mm height) were machined from the homogenized ingots. Uniaxial hot compression tests were conducted using a Gleeble 3800 thermomechanical testing unit (Dynamic Systems Inc., Poestenkill, NY, USA) at strain rates of 0.01, 0.1, 1 and 10 s^−1^ and temperatures of 400, 450, 500 and 550 °C. To minimize the friction between the sample and anvil during test, thin graphite foils were placed on both ends of the samples. Specimens were deformed to a total true strain of 0.8 and then immediately water-quenched to room temperature to retain the deformed microstructure.

## 3. Results and Discussion

### 3.1. Effect of Cu Content on Flow Stress Behavior

**Figure 1:**
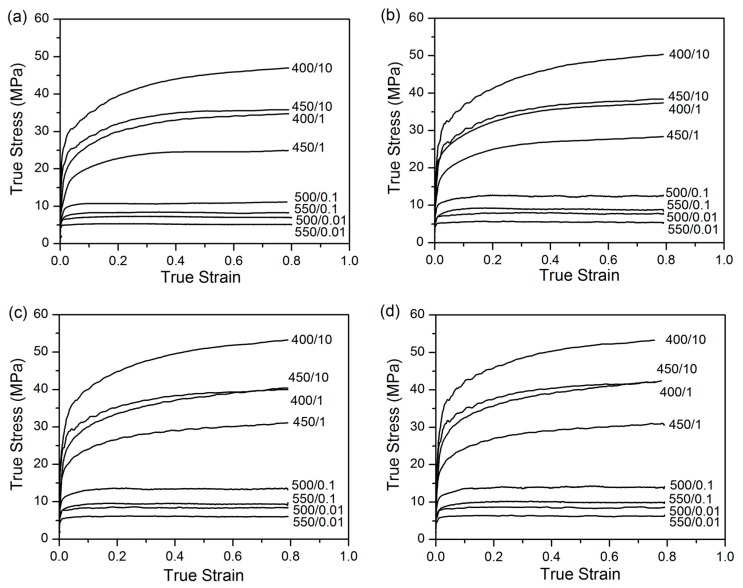
Typical true stress-true strain curves of: (**a**) base alloy; (**b**) 0.05% Cu; (**c**) 0.18% Cu and (**d**) 0.31% Cu.

Hot compression tests of four Al-0.12Fe-0.1Si alloys with various levels of Cu were conducted at different strain rates (0.01 to 10 s^−1^) and temperatures (400 to 550 °C). Figure 1 illustrates the resulting series of true stress-true strain curves obtained during hot deformation. In general, the peak flow stress was followed by a steady state region. However, in some cases, the flow stress continued to increase until the end of straining. The former case occurs when dynamic softening is in balance with work hardening, while the latter phenomenon is indicative of work hardening being stronger than dynamic softening during deformation. Both flow behaviors are normal characteristics of hot working where dynamic recovery (DRV) is the dominant softening mechanism [25,26]. Flow stress increased with increasing strain rate and decreasing deformation temperature for all alloys studied, which is in agreement with previously reported results [9,27,28]. Furthermore, flow stress significantly increased with increasing Cu content.

**Figure 2:**
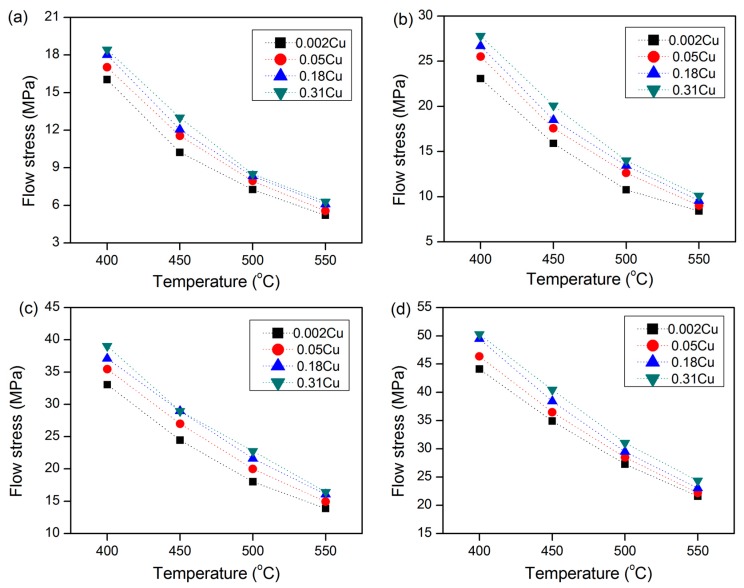
Effect of Cu content on flow stress of Al-0.12Fe-0.1Si alloy at strain of 0.4; (**a**) $\dot{\varepsilon }$ = 0.01 s^−1^; (**b**) $\dot{\varepsilon }$ = 0.1 s^−1^; (**c**) $\dot{\varepsilon }$ = 1 s^−1^ and (**d**) $\dot{\varepsilon }$ = 10 s^−1^.

Figure 2 presents the evolution of flow stress with varying amounts of Cu at a true strain of 0.4 as a function of temperature at different strain rates. It is evident that increasing the Cu content increases the flow stress over the applied range of deformation conditions. For example, at a given deformation condition (*T* = 400 °C, $\dot{\varepsilon }$ = 1 s^−1^), increasing the Cu level from 0.002% to 0.05%, 0.18%, and 0.31% increased the flow stress from 33 to 35.5, 37 and 39 MPa, respectively. These results indicate that the addition of Cu gradually enhances the deformation resistance of the dilute Al-Fe-Si alloy. As Cu has a relatively high solid solubility in aluminum (5.7% at 548 °C [6]), all of the added Cu up to 0.31% is expected to be in the solid solution after homogenization and at the deformation temperature. Microstructural examination of the Cu-containing deformed samples confirmed that Cu did not form any precipitates or dispersoids and that all of the added Cu remained in solid solution (Figures not shown here—more detailed information in Ref. 29). The Cu solute atoms interact with mobile dislocations and retard the dynamic recovery, which leads to significant increases in flow stress during hot deformation [29,30,31].

### 3.2. Development of an Artificial Neural Network Model

**Figure 3:**
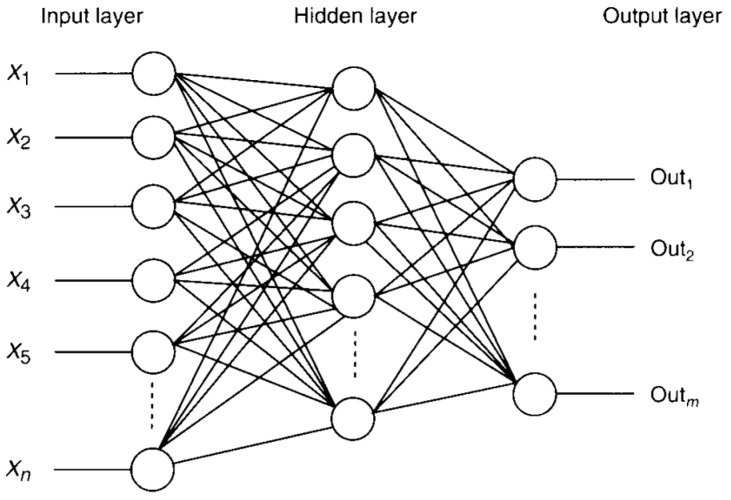
The architecture of the artificial neural network (ANN) model.

A multilayer perceptron (MLP) based feed-forward artificial neural network with a back-propagation (BP) learning algorithm was employed to study the high-temperature flow behavior of Al-0.12Fe-0.1Si-Cu alloys. A general scheme of the three layer network with one hidden layer is given in Figure 3.

A differentiable logistic sigmoid function, given by Equation (1), was employed as the activation function in the present model:

(1) $$F\left(x\right)=\frac{1}{1+\exp\left(-x\right)}$$

In this study, the input parameters of the neural network are: Cu content, strain (ε), temperature (*T*) and strain rate ($\dot{\varepsilon }$). The output is flow stress (σ). A total of 960 experimental data points were selected from the true stress–true strain curves (with an interval of 0.05 between true strains of 0.05 and 0.75) and were employed to train and test the ANN model. To ensure the learning efficiency of the algorithm and prevent a specific factor from dominating learning for the model, both input and output data were normalized within the range of 0–1. The following equation is widely utilized for unification [19,32]:

(2) $$X^{\prime }=0.1+0.8\times \left(\frac{X-X_{\text{min}}}{X_{\text{max}}-X_{\text{min}}}\right)$$

where *X* is the original data, *X*_min_ and *X*_max_ are the minimum and maximum value of *X*, and *X*’ is the unified data corresponding to *X*. In this work, Equation (2) was utilized to unify the Cu level, temperature and stress values. The strain rate changes greatly from 0.01 to 10 s^−1^; therefore, the normalized value of $\dot{\varepsilon }$ is too small to learn by ANN, and the following equation was adopted to unify its value [19].

(3) $${\dot{\varepsilon }}^{'}=0.1+0.8\times \left(\frac{\log\dot{\varepsilon }\;-\log{\dot{\varepsilon }}_{\text{min}}}{\log{\dot{\varepsilon }}_{\text{max}}-\log{\dot{\varepsilon }}_{\text{min}}}\right)$$

**Figure 4:**
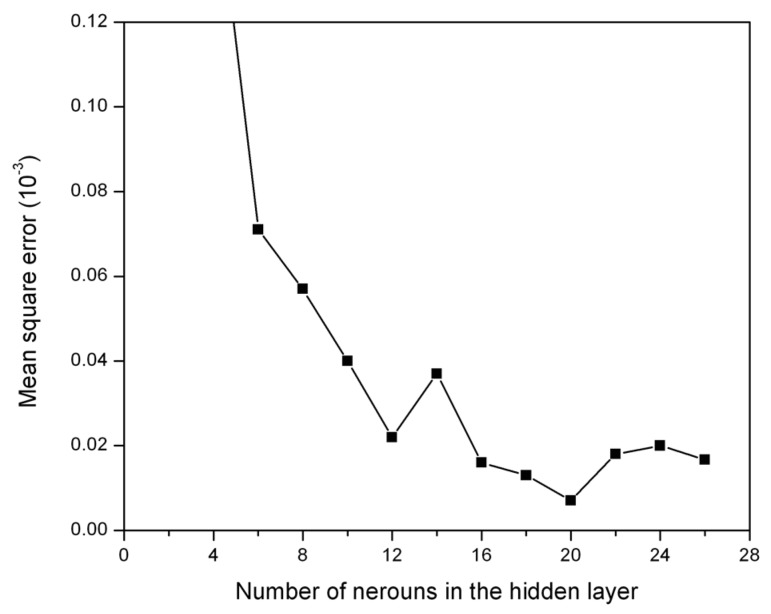
Performance of the network with different numbers of neurons in the hidden layer.

The ε values are already in the range of 0 to 1 and therefore do not need further unification. The three layer network with one hidden layer was found to be fully sufficient for this study. To define the number of neurons in the hidden layer, mean square error (MSE) values obtained using Equation (4) were employed as the indices to evaluate the capability of a given network [22]:

(4) $$\text{MSE}=\frac{1}{N}\overset{N}{\underset{i=1}{\sum }}{\left(E_{i}-P_{i}\right)}^{2}$$

where *E_i_* is the experimental value and *P_i_* is the predicted value obtained from the ANN. *N* is the total number of data employed in the study. Neurons in the hidden layer were varied from 4 to 26. It was observed that a network with 20 hidden neurons gives a minimal MSE with very good correlation (Figure 4).

The datasets obtained from compression tests were randomly divided into two groups: 80% of the datasets were used to train the network model (training dataset), and the remaining 20% were applied to test the model performance (test dataset). The work was accomplished by using the neural network toolbox available with MATLAB 7.6.0.324 software (MathWorks, Natick, MA, USA).

#### 3.2.1. Effect of Cu Addition

**Figure 5:**
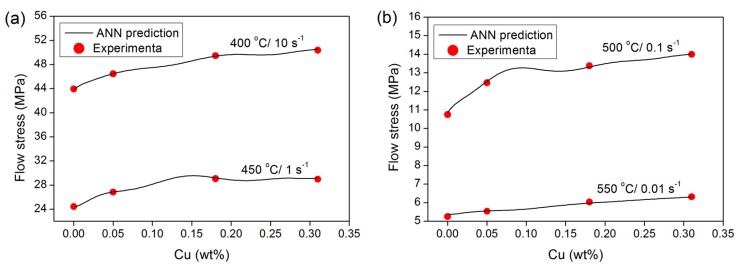
Effect of Cu content on the flow stress at a strain of 0.4 (**a**) for 400 °C/10 s^−1^ and 450 °C/1 s^−1^; and (**b**) for 500 °C/0.1 s^−1^ and 550 °C/0.01 s^−1^.

The ANN model was employed to evaluate the effect of Cu content on high-temperature flow behavior. Figure 5 shows the variations in flow stress at a true strain of 0.4 as a function of Cu content for various deformation conditions. It is clear that the predicted results agree well with the experimental data. The increase in flow stress with the addition of Cu is attributed to the solid solution strengthening effect of Cu, which is a result of interactions between the mobile dislocations and the solute atoms. The presence of Cu solutes increases the high-temperature flow stress and decreases the dynamic recovery rate due to the solute drag effect on dislocation movement [6,29,30,31]. With an increase in Cu content, the Cu solutes produced a stronger solute-drag effect and further decreased the dynamic recovery level and subsequently increased flow stress.

#### 3.2.2. Effect of Temperature

**Figure 6:**
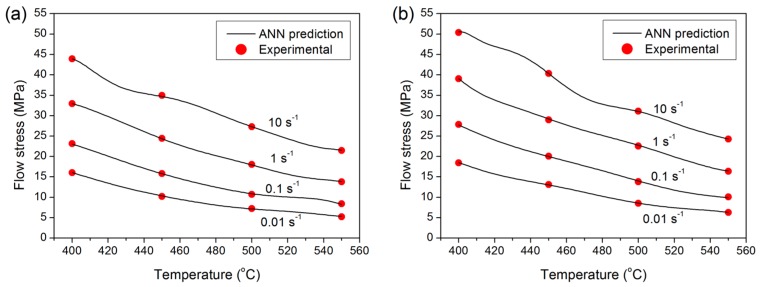
Effect of deformation temperature on the flow stress of experimental alloys at a strain of 0.4; (**a**) base alloy; and (**b**) 0.31% Cu.

The effects of deformation temperature on high-temperature flow behavior were studied using the developed ANN model. Representative results are illustrated in Figure 6. The simulated flow stress values exhibited excellent agreement with the corresponding measured data for a range of strain rates and Cu contents. The flow stress decreased over the range of tested strain rates with increasing deformation temperature for all of the alloys investigated. It is well known that dynamic softening is a thermally activated process [7,33]. Hence, as the temperature increases, the available thermal activation energy increases, which leads to a higher level of dynamic softening and a reduced flow stress.

#### 3.2.3. Effect of Strain Rate

**Figure 7:**
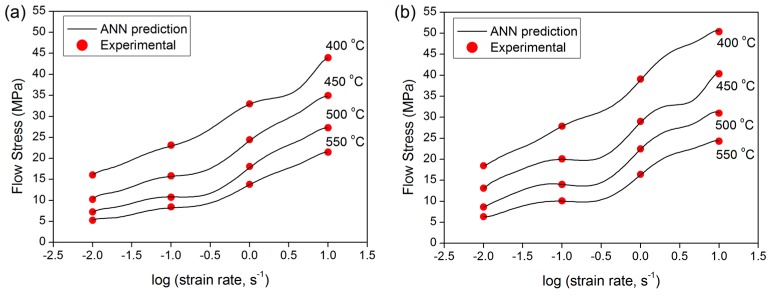
Effect of deformation strain rate on the flow stress of experimental alloys at a strain of 0.4; (**a**) base alloy; and (**b**) 0.31% Cu.

Figure 7 shows plots of σ versus log$\dot{\varepsilon }$ comparing the experimental data with the ANN predicted values at varied forming temperatures. The trained network is able to accurately predict the influence of strain rate on flow stress. Increasing the strain rate results in increased flow stress, as expected. Increasing the strain rate at a given temperature causes an increased dislocation multiplication rate and increased formation of tangled dislocation structures that act as barriers to dislocation movement. Consequently, the flow stress is augmented [7,33].

#### 3.2.4. Assessment of the Proposed Model

In this study, the K-fold cross-validation has been employed to evaluate the performance of the designed network. The cross-validation is an evaluation method that estimates generalization error based on resampling [34,35,36,37], and the K-fold cross-validation method is one of the most commonly used cross-validation techniques to evaluate the architecture and performance of an ANN [34]. In this technique, the dataset is randomly split into K mutually exclusive subsets (the folds) of approximately equal size. K-1 subsets are employed to train the ANN model, and remaining one subset is used to evaluate the network performance. However, the training and validation processes are repeated K times with each subset used once as the validation set [34,35,36,37]. In general, five or 10 fold cross-validations are recommended as a good compromise [34,35].

**Figure 8:**
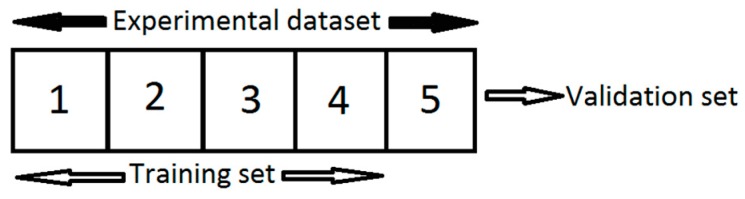
Partitioning of experimental data for K = 5.

In the present work, K was set to five and the datasets obtained from the compression tests were randomly divided into five equal size subsets (each containing 20% of the datasets). Subsequently, five iterations of training and validation were performed in such way that in each iteration a different fold of the data was held out for validation while the remaining four folds were used for training (Figure 8). The performance of the designed network on each validation fold was evaluated using mean square error (MSE, Equation (4)), and the average absolute relative error (AARE), expressed as [22,32]:

(5) $$\text{AARE}\left(\%\right)=\left(\frac{1}{N}\overset{N}{\underset{i=1}{\sum }}\left|\frac{E_{i}-P_{i}}{E_{i}}\right|\right)\times 100$$

where *E_i_* and *P_i_* have the same meaning as stated earlier. The obtained results for each validation folds are given in Table 2. The MSEs and AAREs that obtained for each fold are then averaged to produce a single value for a network design (Table 2). As can be seen, the average values of the MSE and AARE show excellent generalization capability of the designed network over the full range of data.

**Figure 9:**
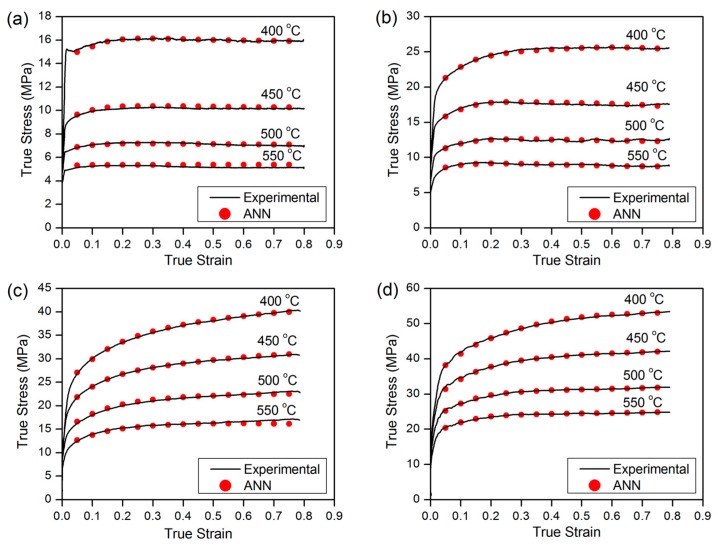
Comparison between predicted ANN model and measured flow stress curves of (**a**) base alloy at $\dot{\varepsilon }$ = 0.01 s^−1^; (**b**) 0.05% Cu alloy at $\dot{\varepsilon }$ = 0.1 s^−1^; (**c**) 0.18% Cu alloy at $\dot{\varepsilon }$ = 1 s^−1^; and (**d**) 0.31% Cu alloy at $\dot{\varepsilon }$ = 10 s^−1^.

Figure 9 illustrates the predicted and experimental flow curves of Al-0.12Fe-0.1Si-Cu alloys for selected deformation conditions. Excellent agreement between predicted and experimental values over the full range of data also reveals the capability of the proposed ANN model to predict hot deformation behavior. Therefore, the model appears to offer significant potential compared to the phenomenological or analytical approaches, which are typically utilized to predict the flow behavior of a given chemical composition.

#### 3.2.5. Sensitivity Analysis

A sensitivity analysis was carried out to statistically assess the contributions of the input variables in the neural network. Although different methods can be used to quantify the relative importance of input parameters [38,39], the algorithm proposed by Garson [40] is found to be the most robust method. This method includes partitioning hidden-output connection weights into components that are associated with each input neuron using absolute values of connection weights (see Appendix A).

**Figure 10:**
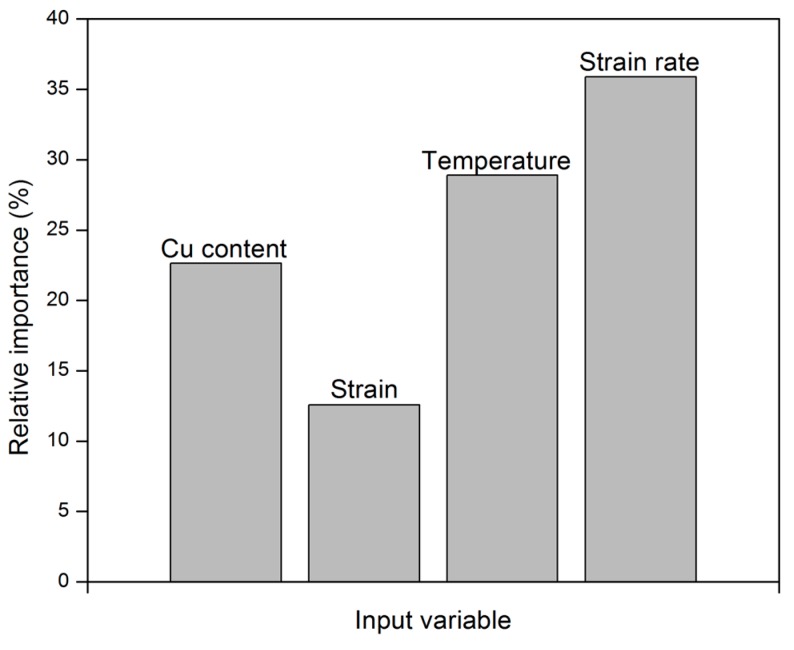
Relative importance of individual input parameters.

Figure 10 shows the relative importance of Cu content and deformation parameters on the flow stress of Al-0.12Fe-0.1Si-Cu alloys. The results revealed that both the strain rate and forming temperature have the most significant effect on the flow stress of the investigated alloys, while strain has a minimal effect. The contribution of strain arises primarily at high Z conditions where the rate of work hardening is greater than dynamic softening. At low Z conditions, the flow stress generally remained constant with changes in strain. The Cu content exhibits a moderate influence on the flow behavior of Al-0.12Fe-0.1Si-Cu alloys.

## 4. Conclusions

A set of uniaxial hot compression tests were carried out on Al-0.12Fe-0.1Si alloys with varied Cu contents at various temperatures (400 °C–550 °C) and strain rates (0.01–10 s^−1^). The results showed that increasing Cu content increases the flow stress over the applied range of deformation conditions due to solid solution strengthening. Based on the experimental results, a three-layer feed-forward artificial neural network model with a back-propagation learning algorithm was developed to predict the high-temperature flow behavior of the Al-0.12Fe-0.1Si-Cu alloys. It was found that the ANN model with one hidden layer consisting of 20 neurons gives the best performance. The simulated results demonstrated excellent agreement with corresponding experimental results. The predictability of the proposed model was also assessed using the K-fold cross-validation method. It is confirmed that the ANN model is an accurate and reliable tool to predict the high-temperature flow behavior of Al-0.12Fe-0.1Si-Cu alloys as a function of alloy composition and deformation variables. Furthermore, sensitivity analysis indicates that both the strain rate and the temperature have the most significant impact on the high-temperature flow stress, while the Cu content exhibits a moderate influence.

## Figures and Tables

**Table 1:** Chemical compositions of alloys (wt %).

Alloys	Si	Fe	Cu	Mn	Cr	Ni	Ti	Co	Zr	V
Base alloy	0.10	0.12	0.002	0.001	0.001	0.007	0.016	0.0003	0.0015	0.012
Al-0.12Fe-0.1Si-**0.05Cu**	0.10	0.12	0.051	0.001	0.001	0.007	0.016	0.0003	0.0014	0.012
Al-0.12Fe-0.1Si-**0.18Cu**	0.11	0.13	0.181	0.001	0.001	0.007	0.015	0.0003	0.0014	0.013
Al-0.12Fe-0.1Si-**0.31Cu**	0.11	0.13	0.31	0.00	0.001	0.007	0.015	0.0003	0.0014	0.012

**Table 2:** The value of mean square error (MSE) and average absolute relative error (AARE) for different validation folds.

Validation Fold	1	2	3	4	5	Average
MSE	0.063	0.046	0.052	0.072	0.059	0.058
AARE (%)	1.36	0.94	1.07	1.63	1.28	1.26

**Figure A1:**
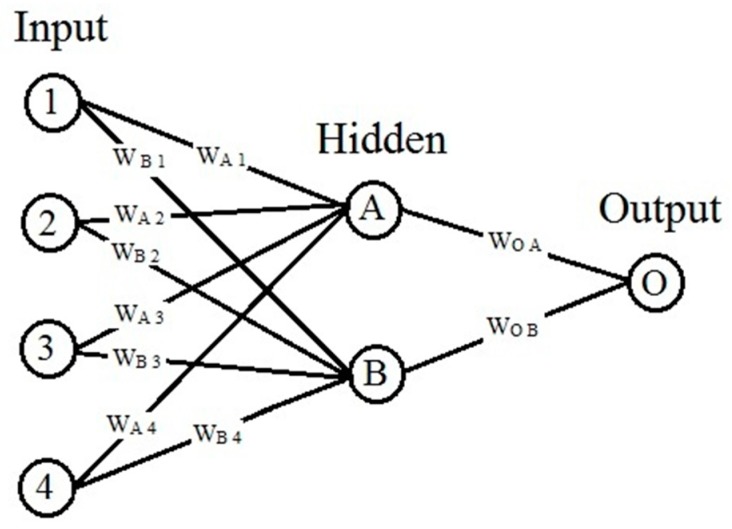
The structure of a 4-2-1 artificial neural network.

## Appendix A. Example Illustrating Garson’s Algorithm

This example describes the procedure to determine the relative importance of input parameters using Garson’s algorithm [38,40].

As an example, consider an ANN with four input neurons, two hidden neurons and one output neuron with the connection weight as shown in Figure A1.

1. Matrix containing input-hidden and hidden-output neuron connection weights.

**Table A1:** Input-hidden and hidden-output neuron connection weights.

Neurons	Hidden A	Hidden B
Input 1	*W*_A1_ = 1.40	*W*_B1_ = 0.82
Input 2	*W*_A2_ = −1.02	*W*_B2_ = 0.62
Input 3	*W*_A3_ = −2.98	*W*_B3_ = 1.04
Input 4	*W*_A4_ = 3.99	*W*_B4_ = −2.26
Output	*W*_OA_ = −3.17	*W*_OB_ = −1.21

2. Contribution of each input neuron to the output via each hidden neuron calculated as the product of the input-hidden connection and the hidden-output connection: e.g., *C*_A1_ = *W*_A1_ × *W*_OA_ = 1.4 × −3.17 = −4.44.

**Table A2:** Contribution of each input neuron to the output via each hidden neuron.

Neurons	Hidden A	Hidden B
Input 1	*C*_A1_ = −4.44	*C*_B1_ = −0.99
Input 2	*C*_A2_ = 3.23	*C*_B2_ = −0.75
Input 3	*C*_A3_ = 9.45	*C*_B3_ = −1.26
Input 4	*C*_A4_ = 12.65	*C*_B4_ = 2.73

3. Relative contribution of each input neuron to the outgoing signal of each hidden neuron: e.g., *r*_A1_ = |*C*_A1_|/(|*C*_A1_| + |*C*_A2_| + |*C*_A3_| + |*C*_A4_|) = 4.44/(4.44 + 3.23 + 9.45 + 12.65) = 0.15 and the sum of input neuron contributions: e.g., *S*_1_ = *r*_A1_ + *r*_B1_ = 0.15 + 0.17 = 0.14.

**Table A3:** Relative contribution of each input neuron to the outgoing signal of each hidden neuron.

Neurons	Hidden A	Hidden B	Sum
Input 1	*r*_A1_ = 0.15	*r*_B1_ = 0.17	*S*_1_ = 0.32
Input 2	*r*_A2_ = 0.11	*r*_B2_ = 0.13	*S*_2_ = 0.24
Input 3	*r*_A3_ = 0.32	*r*_B3_ = 0.22	*S*_3_ = 0.54
Input 4	*r*_A4_ = 0.42	*r*_B4_ = 0.48	*S*_4_ = 0.9

4. Relative importance (RI) of each input variable: e.g., RI_1_ = *S*_1_/(*S*_1_ + *S*_2_ + *S*_3_ + *S*_4_) × 100 = 0.32/(0.32 + 0.24 + 0.54 + 0.9) × 100 = 16%.

**Table A4:** Relative importance of each input variable.

Neurons	Relative Importance (%)
Input 1	16
Input 2	12
Input 3	27
Input 4	45

To determine the relative importance of Cu content and each deformation variable on the flow stress of Al-Fe-Si-Cu alloys, the same calculation method was used for the developed ANN model with 20 neurons on its hidden layer.
